# Evolutive Models, Algorithms and Predictive Parameters for the Progression of Hepatic Steatosis

**DOI:** 10.3390/metabo14040198

**Published:** 2024-04-03

**Authors:** Marinela Sînziana Tudor, Veronica Gheorman, Georgiana-Mihaela Simeanu, Adrian Dobrinescu, Vlad Pădureanu, Venera Cristina Dinescu, Mircea-Cătălin Forțofoiu

**Affiliations:** 1 Doctoral School, University of Medicine and Pharmacy of Craiova, Petru Rareș 2 Str, 200349 Craiova, Romania; marinelasinziana@yahoo.com (M.S.T.); simeanugeorgiana95@gmail.com (G.-M.S.); 2 Department 3 Medical Semiology, University of Medicine and Pharmacy of Craiova, Petru Rareș 2 Str, 200349 Craiova, Romania; vlad.padureanu@umfcv.ro; 3 Department of Thoracic Surgery, University of Medicine and Pharmacy of Craiova, Petru Rareș 2 Str, 200349 Craiova, Romania; 4 Department of Health Promotion and Occupational Medicine, University of Medicine and Pharmacy of Craiova, Petru Rareș 2 Str, 200349 Craiova, Romania; venera.dinescu@umfcv.ro; 5 Department 3 Medical Semiology, University of Medicine and Pharmacy of Craiova, Clinical Municipal Hospital “Philanthropy” of Craiova, 200143 Craiova, Romania; catalin.fortofoiu@umfcv.ro

**Keywords:** hepatic steatosis, evolutive models, algorithms, computational approaches, progression prediction, predictive parameters

## Abstract

The utilization of evolutive models and algorithms for predicting the evolution of hepatic steatosis holds immense potential benefits. These computational approaches enable the analysis of complex datasets, capturing temporal dynamics and providing personalized prognostic insights. By optimizing intervention planning and identifying critical transition points, they promise to revolutionize our approach to understanding and managing hepatic steatosis progression, ultimately leading to enhanced patient care and outcomes in clinical settings. This paradigm shift towards a more dynamic, personalized, and comprehensive approach to hepatic steatosis progression signifies a significant advancement in healthcare. The application of evolutive models and algorithms allows for a nuanced characterization of disease trajectories, facilitating tailored interventions and optimizing clinical decision-making. Furthermore, these computational tools offer a framework for integrating diverse data sources, creating a more holistic understanding of hepatic steatosis progression. In summary, the potential benefits encompass the ability to analyze complex datasets, capture temporal dynamics, provide personalized prognostic insights, optimize intervention planning, identify critical transition points, and integrate diverse data sources. The application of evolutive models and algorithms has the potential to revolutionize our understanding and management of hepatic steatosis, ultimately leading to improved patient outcomes in clinical settings.

## 1. Introduction

Hepatic steatosis, commonly referred to as fatty liver disease, is a condition characterized by the accumulation of fat in the liver cell [1,2]. This excess fat can impair liver function and lead to inflammation, which may progress to more severe conditions such as non-alcoholic fatty liver disease (NAFLD) and non-alcoholic steatohepatitis (NASH) [3]. Hepatic steatosis can result from various factors, including obesity, insulin resistance, excessive alcohol consumption, certain medications, and metabolic disorders [4].

The prevalence of hepatic steatosis has been increasing globally, paralleling the rising rates of obesity and metabolic syndrome [5]. It has become a significant public health concern due to its association with an increased risk of liver-related complications, cardiovascular disease, and other metabolic disorders. Moreover, hepatic steatosis can progress to more advanced stages, potentially leading to liver fibrosis, cirrhosis, and hepatocellular carcinoma.

Given the potential for disease progression and the adverse health outcomes associated with hepatic steatosis, there is a growing need for improved diagnostic methods and predictive parameters to assess the evolution of the condition. This has led to significant research efforts focused on developing evolutive models, algorithms, and predictive parameters to better understand, predict, and manage the progression of hepatic steatosis. By elucidating the factors contributing to disease evolution and identifying effective predictive tools, researchers aim to enhance early detection, risk stratification, and treatment strategies for individuals with hepatic steatosis.

### 1.1. Understanding the Progression of Hepatic Steatosis and the Impact It Has on Public Health

Understanding the progression of hepatic steatosis is significant due to its substantial impact on public health at both individual and population levels. The increasing prevalence of hepatic steatosis, coupled with its potential for progression to more severe liver diseases, underscores the critical need for a comprehensive understanding of its evolution and associated health consequences.

At the individual level, hepatic steatosis can lead to a range of adverse health outcomes, including impaired liver function, inflammation, fibrosis, cirrhosis, and an increased risk of hepatocellular carcinoma [6]. These complications can significantly affect an individual’s quality of life and may necessitate medical interventions such as lifestyle modifications, pharmacological treatments, and, in more advanced cases, liver transplantation [6,7,8]. Therefore, understanding the progression of hepatic steatosis is essential for identifying individuals at higher risk of disease advancement and tailoring appropriate management strategies to mitigate these risks.

On a broader public health scale, hepatic steatosis poses a considerable burden due to its association with other metabolic conditions such as obesity, type 2 diabetes, and cardiovascular disease. The co-occurrence of these conditions can exacerbate the overall disease burden on healthcare systems and contribute to increased healthcare costs [5,9]. Furthermore, the potential for hepatic steatosis to progress to advanced liver diseases places a strain on healthcare resources, necessitating a greater focus on early detection, risk stratification, and the prevention of disease progression.

In addition to the direct health implications, hepatic steatosis also has indirect societal and economic impacts. Individuals with advanced liver diseases stemming from hepatic steatosis may experience impaired productivity, increased absenteeism from work, and reduced overall functional capacity, leading to economic repercussions for both affected individuals and the wider community. Furthermore, the need for extensive medical care and potential complications associated with advanced liver diseases can place a significant economic burden on healthcare systems and society as a whole.

Given these multifaceted implications, understanding the progression of hepatic steatosis is crucial for informing public health policies, preventive strategies, and healthcare resource allocation. By gaining insights into the factors that drive disease progression, identifying high-risk individuals and refining predictive models, public health initiatives can be better tailored to address the evolving landscape of hepatic steatosis and its associated complications. This may involve implementing targeted screening programs, enhancing lifestyle interventions, developing novel treatment approaches, and promoting multidisciplinary collaborations to effectively address the growing burden of hepatic steatosis and its impact on public health.

In our manuscript, we will delve into the utilization of evolutive models and algorithms for predicting the evolution of hepatic steatosis. These computational approaches offer significant potential benefits, enabling the analysis of complex datasets and capturing temporal dynamics to provide personalized prognostic insights. We will discuss how these methods optimize intervention planning, identify critical transition points, and promise to revolutionize our approach to understanding and managing hepatic steatosis progression. Our exploration will highlight the nuanced characterization of disease trajectories facilitated by evolutive models and algorithms, leading to tailored interventions and optimized clinical decision-making.

### 1.2. Current Diagnostic and Predictive Parameters

Conventional methods for diagnosing and predicting the progression of hepatic steatosis encompass a range of diagnostic tests and imaging studies, each with its own strengths and limitations [10]. Additionally, histological analysis through liver biopsy remains a gold standard for accurately assessing the severity of hepatic steatosis and identifying associated liver pathology [11].

1. Liver function tests (LFTs) are routinely used to assess liver health and may indicate the presence of hepatic steatosis, although they are not specific to this condition alone [12]. Elevations in serum levels of alanine aminotransferase (ALT) and aspartate aminotransferase (AST) are commonly observed in hepatic steatosis, indicating liver cell injury or inflammation [11,12]. However, these biomarkers lack specificity and may not necessarily correlate with the severity or progression of the disease. Additionally, serum levels of gamma-glutamyl transferase (GGT) and alkaline phosphatase may be elevated in individuals with hepatic steatosis, providing further biochemical evidence of liver dysfunction [13].
2. Imaging studies play a critical role in the evaluation of hepatic steatosis [14]. Ultrasonography is often used as an initial imaging modality due to its wide availability, cost-effectiveness, and absence of ionizing radiation [14]. While ultrasonography can detect moderate to severe hepatic steatosis based on characteristic patterns of echogenicity, it may be less sensitive in identifying mild cases and can be operator-dependent.
3. Computed tomography (CT) and magnetic resonance imaging (MRI) also offer valuable insights into hepatic steatosis [15]. CT scans can detect hepatic steatosis based on altered liver density, and MRI, particularly using specialized sequences such as proton density fat fraction (PDFF) imaging, offers high sensitivity and specificity for quantifying liver fat content. MRI is especially advantageous in differentiating hepatic steatosis from other liver diseases and can provide accurate assessments of fat distribution within the liver [16].
4. Histological analysis through liver biopsy remains the gold standard for diagnosing and grading hepatic steatosis [11]. It allows for the precise histopathological evaluation of liver tissue, including the extent of fat accumulation, the presence of inflammation, and any concurrent liver pathology. However, liver biopsy is an invasive procedure associated with potential complications and sampling variability, making it less suitable for longitudinal monitoring and large-scale population-based assessments.
  While these conventional diagnostic methods provide valuable information, their limitations have spurred the exploration of alternative and complementary approaches to diagnose and predict the progression of hepatic steatosis. Specifically, there is a growing interest in non-invasive biomarkers, imaging modalities, and predictive models that can enhance the accuracy, accessibility, and longitudinal monitoring of hepatic steatosis.
5. Non-invasive biomarkers, such as the NAFLD fibrosis score and the Fibrosis-4 index, have been developed to assess the likelihood of advanced fibrosis in individuals with NAFLD, including those with hepatic steatosis [17]. These biomarkers incorporate clinical and laboratory parameters to estimate the degree of liver fibrosis, serving as valuable tools for risk stratification and prognostication.
6. In addition, innovative imaging techniques, such as magnetic resonance elastography (MRE), have emerged as promising non-invasive methods for quantifying liver stiffness, a surrogate marker of fibrosis severity [18]. MRE can provide comprehensive assessments of both liver fat content and fibrosis, offering a holistic evaluation of hepatic steatosis and its potential progression to more advanced liver diseases.
  Moreover, predictive models and risk stratification algorithms have been developed to identify individuals with hepatic steatosis who are at higher risk of disease progression [19,20,21]. These models often integrate demographic, clinical, laboratory, and imaging data to predict the likelihood of adverse outcomes, such as the development of NASH or advanced fibrosis. By leveraging machine learning algorithms and longitudinal data, these predictive models aim to guide clinical decision-making and improve patient management strategies.

### 1.3. Highlighting the Limitations and Challenges Associated with the Current Diagnostic and Predictive Parameters, including Issues Related to Accuracy, Sensitivity, and Specificity

The current diagnostic and predictive parameters for hepatic steatosis face several limitations and challenges that impact their accuracy, sensitivity, and specificity. These constraints underscore the need for improved methodologies to more effectively diagnose and predict the progression of this condition.

#### 1.3.1. Lack of Specificity in Liver Function Tests

While liver function tests (LFTs) are commonly employed to assess hepatic steatosis, they lack specificity for this condition alone. Elevations in serum levels of alanine aminotransferase (ALT) and aspartate aminotransferase (AST) can be indicative of liver cell injury or inflammation, but they are not specific to hepatic steatosis and may not reliably correlate with disease severity or progression [22,23]. As a result, LFTs alone may not provide a conclusive diagnosis or accurate prediction of hepatic steatosis outcomes.

#### 1.3.2. Ultrasonography Limitations

Although ultrasonography is widely used for the initial screening of hepatic steatosis due to its accessibility and cost-effectiveness, it has limitations in terms of sensitivity, especially for detecting mild cases of hepatic steatosis [10]. Operator dependence and factors such as body habitus and presence of concomitant liver disease can further hinder the accuracy of ultrasound-based diagnosis and the prediction of hepatic steatosis.

#### 1.3.3. Invasive Nature of Liver Biopsy

Histological analysis through liver biopsy remains the gold standard for diagnosing hepatic steatosis, but it is an invasive procedure associated with inherent risks, including bleeding, pain, and sampling variability [24,25]. These limitations make liver biopsy less suitable for longitudinal monitoring and large-scale population-based assessments of hepatic steatosis progression.

#### 1.3.4. Need for Improved Non-Invasive Biomarkers

While non-invasive biomarkers such as the NAFLD fibrosis score and the Fibrosis-4 index provide valuable risk stratification for advanced fibrosis in individuals with NAFLD, there is a need for more accurate and specific biomarkers dedicated to diagnosing and predicting the progression of hepatic steatosis itself [26,27]. Current biomarkers may lack the precision required to differentiate between different stages of hepatic steatosis and to predict individual disease trajectories.

#### 1.3.5. Imaging Modalities and Limitations

While advanced imaging modalities such as CT, MRI, and magnetic resonance elastography (MRE) offer enhanced capabilities for assessing hepatic steatosis and fibrosis, challenges related to accessibility, cost, and standardized interpretation exist [28]. Furthermore, the interpretation of imaging findings can be affected by factors such as concurrent liver pathology and the need for expertise in radiological interpretation.

#### 1.3.6. Predictive Model Complexities

Predictive models and risk stratification algorithms that aim to identify individuals at higher risk of hepatic steatosis progression rely on complex data integration and algorithm development [29,30]. Challenges may arise in ensuring the accuracy, generalizability, and interpretability of these models across diverse patient populations and healthcare settings.

#### 1.3.7. Dynamic Nature of Hepatic Steatosis

Hepatic steatosis is a dynamic condition influenced by multiple factors, including genetic predisposition, lifestyle behaviors, and comorbidities such as obesity and diabetes. The complexity of these interactions presents challenges in accurately predicting disease progression and individual response to interventions.

Addressing these limitations and challenges requires ongoing research and innovation to develop more accurate, sensitive, and specific diagnostic and predictive parameters for hepatic steatosis. Improved biomarkers, imaging technologies and predictive models tailored to the unique characteristics of hepatic steatosis can enhance early detection, prognostication, and personalized management strategies for individuals at risk of progression to advanced liver disease. Additionally, a comprehensive understanding of the underlying pathophysiology and natural history of hepatic steatosis is essential for refining diagnostic and predictive approaches.

## 2. Method

### 2.1. Evolutive Models and Algorithms

#### 2.1.1. The Concept of Evolutive Models and Algorithms in the Context of Hepatic Steatosis Progression

Evolutive models and algorithms play a crucial role in understanding and predicting the progression of hepatic steatosis. In the context of this condition, evolutive models and algorithms refer to computational approaches that account for the dynamic nature of hepatic steatosis, taking into consideration the temporal evolution of the disease and its interactions with various influencing factors. These models and algorithms involve capturing the complex, multifactorial aspects of hepatic steatosis progression, incorporating longitudinal data, individual variability, and the dynamic interplay of biological, environmental, and behavioral determinants.

##### Longitudinal Data Integration

Evolutive models and algorithms aim to utilize longitudinal data to track disease progression over time, enabling the identification of patterns, trajectories, and transitions in hepatic steatosis severity. By incorporating data from repeated measurements, these models can provide insights into the natural history of the condition, including the rate of progression, fluctuations in disease activity, and responses to interventions.

##### Dynamic Interactions

Hepatic steatosis is influenced by a multitude of factors, including genetic predisposition, lifestyle behaviors, metabolic comorbidities, and environmental exposures [31]. Evolutive models and algorithms aim to take into account the dynamic interactions among these factors, recognizing that the progression of hepatic steatosis is not solely determined by static risk factors but is also shaped by evolving physiological, genetic, and environmental influences.

##### Personalized Predictive Capabilities

By considering individual variability and dynamic changes in risk factors, evolutive models and algorithms have the potential to generate personalized predictions of hepatic steatosis progression. These models can adapt to an individual’s changing risk profile over time, offering tailored prognostic assessments and informing personalized management strategies.

##### Adaptive Learning and Updating

Evolutive models and algorithms aim to adapt to new data and emerging knowledge, allowing for the ongoing refinement and updating of predictive capabilities. As new information becomes available, these models can incorporate it to enhance their accuracy and relevance in predicting hepatic steatosis progression.

##### Contextual Sensitivity

Hepatic steatosis progression can vary across different populations, subgroups, and clinical contexts. Evolutive models and algorithms aim to account for this contextual sensitivity, recognizing that the determinants and trajectories of disease progression may differ based on demographic, genetic, and clinical factors.

##### Intervention Planning and Optimization

In the context of hepatic steatosis, evolutive models and algorithms can be leveraged to simulate the potential impact of interventions on disease progression. By integrating data on treatment outcomes and lifestyle modifications, these models can inform the optimization of intervention strategies, including the timing and intensity of therapeutic interventions to mitigate hepatic steatosis progression.

##### Prognostic Assessments

Evolutive models and algorithms have the capacity to provide prognostic assessments that go beyond static risk prediction. By capturing the dynamic changes in risk factors, disease activity and responses to interventions, these models can offer more nuanced prognostic insights, enhancing the ability to anticipate individual outcomes and identify critical periods for intervention.

The application of evolutive models and algorithms in the context of hepatic steatosis progression represents a paradigm shift towards a more dynamic, personalized, and comprehensive approach to understanding and predicting the natural history of the condition. These computational tools have the potential to advance our ability to characterize disease trajectories, tailor interventions, and optimize clinical decision-making in hepatic steatosis management. Additionally, they offer a framework for integrating diverse sources of data and knowledge to create a more holistic and insightful understanding of hepatic steatosis progression, ultimately leading to improved patient outcomes.

### 2.2. Patient-Centered Outcomes

The integration of evolutive models, algorithms, and predictive parameters in understanding and predicting the progression of hepatic steatosis represents a significant step forward in personalized healthcare. By utilizing computational approaches to analyze complex datasets and capture temporal dynamics, we can tailor interventions and enhance clinical decision-making for improved patient outcomes. These tools enable a more nuanced characterization of disease trajectories, helping clinicians identify critical transition points and optimize treatment plans.

In the realm of patient-centered outcomes research, there is a growing emphasis on aligning predictive models with patient preferences, quality of life measures, and individualized treatment goals. By incorporating these aspects into the development and application of evolutive models and algorithms, we can ensure that the predictions generated are not only clinically relevant but also meaningful to patients. This patient-centered approach helps bridge the gap between data-driven insights and personalized care, ultimately leading to better outcomes and improved patient satisfaction.

The potential benefits of incorporating patient-centered outcomes research into the utilization of evolutive models and algorithms for hepatic steatosis progression are manifold. By focusing on patient preferences and quality of life measures, we can better understand the impact of the disease on individuals and tailor interventions to meet their specific needs. This approach helps shift the focus from purely clinical outcomes to the holistic well-being of the patient, promoting a more comprehensive and compassionate healthcare experience.

Furthermore, by considering individualized treatment goals in the predictive modeling process, we can enhance the relevance and applicability of the generated predictions. This ensures that the insights provided by evolutive models and algorithms are not only accurate from a clinical perspective but also meaningful and actionable for patients. In doing so, we empower both healthcare providers and patients to make informed decisions that align with their individual goals and preferences, leading to more effective and patient-centered care.

In summary, the integration of patient-centered outcomes research with evolutive models, algorithms, and predictive parameters for hepatic steatosis progression represents a significant advancement in healthcare. By emphasizing patient preferences, quality of life measures, and individualized treatment goals, we can enhance the relevance, impact, and applicability of predictive models, ultimately leading to improved patient outcomes and a more personalized approach to care.

## 3. Results

### 3.1. The Potential Benefits of Utilizing Evolutive Models and Algorithms for Predicting the Evolution of Hepatic Steatosis, including Their Ability to Analyze Complex Datasets and Identify Patterns over Time

The utilization of evolutive models and algorithms for predicting the evolution of hepatic steatosis offers several potential benefits, particularly in the context of analyzing complex datasets and identifying patterns over time. These computational approaches aim to have the capacity to significantly enhance our understanding of disease progression, provide personalized prognostic insights, and optimize clinical decision-making. Here are some of the potential benefits.

#### 3.1.1. Capturing Temporal Dynamics

Evolutive models and algorithms are well-suited for capturing the temporal dynamics of hepatic steatosis progression. By incorporating longitudinal data, these models can extract temporal patterns, trends, and fluctuations in disease activity, enabling a more comprehensive understanding of how hepatic steatosis evolves over time. This capacity to capture temporal dynamics is crucial for characterizing the natural history of the disease and identifying critical periods of progression or regression.

#### 3.1.2. Incorporating Multifactorial Interactions

Hepatic steatosis is influenced by a multitude of interconnected factors, including genetic, environmental, metabolic, and behavioral determinants [32]. Evolutive models and algorithms aim to analyze and integrate complex, multifactorial datasets, allowing for the identification of interactions and dependencies among diverse variables that contribute to hepatic steatosis progression. This capability is essential for unraveling the intricate web of factors that influence disease evolution, providing insights into the interplay of risk factors and their impact on disease trajectory.

#### 3.1.3. Personalized Prognostic Insights

The ability of evolutive models and algorithms aim to generate personalized prognostic insights representing a significant benefit for predicting the evolution of hepatic steatosis. By accounting for individual variability and capturing dynamic changes in risk factors, these models can offer tailored predictions of disease progression for specific patients. This personalized approach enhances the accuracy and relevance of prognostic assessments, empowering clinicians to better anticipate a patient’s future risk of hepatic steatosis progression and tailor interventions accordingly.

#### 3.1.4. Predictive Power for Intervention Planning

Evolutive models and algorithms aim to have the potential to inform the planning and optimization of interventions for managing hepatic steatosis progression. By simulating the impact of different interventions over time, these models can help predict the effectiveness of therapeutic strategies, lifestyle modifications, or pharmacological interventions in mitigating hepatic steatosis progression. This predictive power can guide clinicians in selecting the most effective and timely interventions for individual patients, optimizing disease management, and potentially preventing disease exacerbation.

#### 3.1.5. Identification of Critical Transition Points

Through the analysis of complex datasets and the detection of temporal patterns, evolutive models and algorithms can aid in the identification of critical transition points in hepatic steatosis progression. These transition points may signify shifts in disease activity, the onset of complications, or the response to interventions. By identifying such critical junctures, these models can help clinicians recognize opportune moments for intervention, enabling proactive management strategies to mitigate disease progression and improve patient outcomes.

#### 3.1.6. Integration of Diverse Data Sources

Evolutive models and algorithms have the capacity to integrate diverse sources of data, including clinical, genetic, imaging, and omics data, to provide a more comprehensive analysis of hepatic steatosis progression. This integration enables a holistic understanding of the disease’s evolution, allowing for the incorporation of multiple layers of information to elucidate the complex mechanisms and trajectories underlying hepatic steatosis progression.

In summary, the potential benefits of utilizing evolutive models and algorithms for predicting the evolution of hepatic steatosis are vast. These computational approaches offer the capability to analyze complex datasets, capture temporal dynamics, provide personalized prognostic insights, optimize intervention planning, identify critical transition points, and integrate diverse sources of data. As a result, they have the potential to revolutionize our approach to understanding and managing hepatic steatosis progression, ultimately leading to improved patient care and outcomes in the clinical setting.

## 4. Discussion

### 4.1. Predictive Parameters and Biomarkers

The identification and validation of predictive parameters and biomarkers for assessing the progression of hepatic steatosis represent a critical area of research aimed at improving the accuracy, specificity, and accessibility of diagnostic and prognostic tools. Several parameters and biomarkers have demonstrated promise in this regard, offering insights into disease activity, severity, and the risk of progression. Here, we will discuss some of the key predictive parameters and biomarkers that have shown potential for assessing the progression of hepatic steatosis.

#### 4.1.1. Liver Enzymes and Function Tests

The serum levels of liver enzymes such as alanine aminotransferase (ALT) and aspartate aminotransferase (AST) have been widely studied as potential predictive parameters for hepatic steatosis progression. Elevations in ALT and AST levels are commonly associated with liver injury and inflammation, and their correlation with hepatic steatosis severity has been investigated [33].

Additionally, other liver function tests, such as gamma-glutamyl transferase (GGT) and alkaline phosphatase, have been explored as potential markers of hepatocellular injury and dysfunction in the context of hepatic steatosis progression [34].

#### 4.1.2. Imaging-Based Biomarkers

Advanced imaging modalities, including magnetic resonance imaging (MRI) and spectroscopy (MRS), have been utilized to identify and validate imaging-based biomarkers for assessing hepatic steatosis progression. These techniques enable the non-invasive quantification of hepatic fat content and the characterization of fat distribution within the liver [35].

Parameters derived from imaging studies, such as proton density fat fraction (PDFF) measured by MRI, have demonstrated strong correlations with hepatic steatosis severity and have shown potential for monitoring disease progression over time [36].

#### 4.1.3. Serum Biomarkers of Lipid Metabolism and Inflammation

Various serum biomarkers related to lipid metabolism, insulin resistance, and inflammation have been investigated as potential predictors of hepatic steatosis progression. Examples include adiponectin, leptin, resistin, and cytokines such as interleukin-6 (IL-6) and tumor necrosis factor-alpha (TNF-α) [17].

These biomarkers reflect dysregulated metabolic pathways and inflammatory processes associated with hepatic steatosis, offering insights into the underlying pathophysiology and potential indicators of disease progression [17].

#### 4.1.4. Non-Invasive Fibrosis Markers

As hepatic steatosis can progress to non-alcoholic steatohepatitis (NASH) and advanced fibrosis, non-invasive fibrosis markers have been evaluated as predictive parameters for disease progression. Biomarkers such as the Fibrosis-4 (FIB-4) index and the NAFLD fibrosis score have been developed to assess the risk of advanced fibrosis in patients with hepatic steatosis [37].

These indices incorporate parameters such as age, AST, ALT, and platelet count to estimate the likelihood of advanced fibrosis, providing valuable prognostic information regarding disease progression [38].

#### 4.1.5. Omics-Based Biomarkers

Advances in omics technologies, including genomics, transcriptomics, proteomics, and metabolomics, have opened new avenues for the identification of molecular biomarkers associated with hepatic steatosis progression [39]. These biomarkers offer insights into the molecular mechanisms driving disease evolution and can serve as predictors of disease severity and progression.

For example, gene expression profiles associated with lipid metabolism, inflammatory pathways, and fibrogenesis have been investigated as potential molecular biomarkers for assessing the risk of hepatic steatosis progression and the development of NASH and fibrosis [40,41].

#### 4.1.6. Novel Serum Markers and Panels

Several novel serum markers and multiparametric panels have been proposed as predictive tools for assessing the progression of hepatic steatosis. These may include combinations of traditional biomarkers, novel protein markers, or computational algorithms that integrate multiple parameters to enhance predictive accuracy [42,43].

Validation studies for these predictive parameters and biomarkers have aimed to assess their performance in predicting hepatic steatosis progression, disease severity, the risk of complications, and response to interventions. Robust validation is essential to establish the clinical utility and reliability of these biomarkers, ensuring their accuracy and reproducibility across diverse patient populations and clinical settings.

Overall, the identification and validation of predictive parameters and biomarkers for assessing the progression of hepatic steatosis represent a promising avenue for enhancing diagnostic and prognostic capabilities in the management of this prevalent liver condition. By leveraging these biomarkers, clinicians can potentially improve risk stratification, tailor interventions, and monitor disease progression with greater precision, ultimately contributing to more effective patient care and the development of targeted therapeutic strategies.

### 4.2. The Reliability and Clinical Significance of These Predictive Parameters, Considering Their Ability to Predict Disease Progression, Severity and Potential Outcomes

The reliability and clinical significance of predictive parameters for hepatic steatosis are essential considerations with regards to their ability to predict disease progression, severity, and potential outcomes. Evaluating the predictive parameters involves assessing their sensitivity, specificity, and positive and negative predictive values, as well as their ability to discriminate between different stages of hepatic steatosis. Furthermore, the clinical significance of these parameters is determined by their capacity to provide actionable insights that can guide patient management and improve outcomes.

Non-invasive biomarkers, such as serum markers of liver function, inflammation, and fibrosis, have been explored for their potential in predicting the progression and severity of hepatic steatosis. While these biomarkers offer the advantage of non-invasiveness, their reliability and clinical significance depend on their ability to accurately reflect the pathological processes associated with hepatic steatosis [43]. Therefore, the validation of these biomarkers through robust clinical studies is crucial to establish their reliability and clinical significance.

Advanced imaging modalities, including CT, MRI, and magnetic resonance elastography, also play a significant role in predicting the progression and severity of hepatic steatosis. These modalities can provide detailed information about hepatic fat content, inflammation, fibrosis, and liver stiffness, which are important factors in assessing disease progression and severity. However, the reliability and clinical significance of these imaging parameters depend on their accuracy in detecting and quantifying hepatic steatosis, as well as their ability to predict clinically relevant outcomes such as liver-related complications and mortality [44].

In the context of predictive models and algorithms, their reliability and clinical significance lie in the ability to integrate multiple parameters, such as biomarkers, imaging findings, and clinical data, to provide accurate predictions of disease progression, severity, and outcomes. These models should undergo rigorous validation to ensure their reliability and clinical significance in diverse patient populations. Furthermore, the integration of evolving data, such as longitudinal changes in biomarker levels and imaging findings, is essential to enhance the predictive capabilities of these models and algorithms.

Overall, the reliability and clinical significance of predictive parameters for hepatic steatosis depend on their accuracy, reproducibility, and ability to predict clinically meaningful outcomes. Robust validation studies, including longitudinal cohorts and outcome-based assessments, are crucial to establish the reliability and clinical significance of these parameters. Furthermore, the incorporation of these predictive parameters into clinical practice should lead to improved risk stratification, patient management, and ultimately, better outcomes for individuals with hepatic steatosis.

A study by Sorino P. provides compelling evidence of the practical effectiveness and reliability of the proposed models and algorithms in real-world clinical settings. By employing a cohort of 2970 subjects and utilizing cross-validation techniques, the study evaluated three distinct models incorporating various predictors for NAFLD diagnosis. The results demonstrated that the support vector machine (SVM) algorithm consistently outperformed other algorithms, achieving high accuracy rates across all models. Notably, Model 3 exhibited the highest accuracy at 77%. These findings suggest that machine learning, particularly the SVM algorithm, holds promise in accurately diagnosing NAFLD, thereby potentially reducing healthcare costs and improving patient outcomes. The simplicity and accessibility of SVM parameters make it a valuable tool for clinical NAFLD screening [45].

The study on the computer-aided diagnosis technique for fatty liver disease (FLD) demonstrates its practical effectiveness and reliability in real-world clinical settings. Using machine learning algorithms and a voting-based classifier, the technique categorizes liver tissues as fatty or normal based on ultrasound image features. The study makes several significant contributions: it achieves liver image classification without segmentation, utilizes a comprehensive dataset of 26 features, and employs a Gray-Level Co-Occurrence Matrix (GLCM) and First-Order Statistics (FOS) for feature extraction. Validation trials of the voting-based classifier and J48 algorithm on the dataset yielded impressive results, with a true positive rate of 94.28%, a true negative rate of 97.14%, a false positive rate of 5.71%, and a false negative rate of 2.85%. Precision, sensitivity, specificity, and F1-score were also high, ranging from 94.28% to 97.05%. The voting-based classifier achieved an accuracy of 95.71%, outperforming the J48 algorithm’s accuracy of 93.12%. These findings demonstrate the robustness and effectiveness of the proposed technique, surpassing previous research works in performance and accuracy. They underscore its potential as a reliable tool for the early detection and diagnosis of fatty liver disease in clinical practice [46].

### 4.3. Exploring Key Considerations in Evaluating Hepatic Steatosis Progression Prediction Models

In the field of hepatic steatosis progression prediction, a comparative analysis of different predictive models and parameters can offer valuable insights into their performance, applicability, and potential for integration. We have delved deeper into some key aspects to consider when evaluating and comparing these predictive tools.

#### 4.3.1. Performance Metrics

When comparing predictive models, it is essential to assess their performance using relevant metrics such as sensitivity, specificity, accuracy, and area under the receiver operating characteristic curve (AUC-ROC). These metrics provide quantitative measures of how well the models can predict the progression of hepatic steatosis.

#### 4.3.2. Model Complexity

Consider the complexity of the predictive models in terms of the number of parameters, computational requirements, and interpretability. Simple models may be easier to implement and interpret but could potentially lack the predictive power of more complex models.

#### 4.3.3. Data Requirements

Evaluate the data requirements of each predictive model, including the types and volume of data needed for training and validation. Models that can effectively utilize diverse data sources and handle missing data may have a broader applicability in real-world clinical settings.

#### 4.3.4. Temporal Dynamics

Assess the ability of predictive models to capture temporal dynamics in hepatic steatosis progression. Models that can account for changes over time and predict future states of the disease may offer more personalized and proactive insights for intervention planning.

#### 4.3.5. Integration Potential

Consider how easily the predictive models can be integrated into existing clinical workflows and decision-making processes. Models that seamlessly integrate with electronic health records, imaging systems, and other healthcare technologies may facilitate more efficient and effective patient care.

#### 4.3.6. Clinical Utility

Ultimately, evaluate the clinical utility of the predictive models in terms of their impact on patient outcomes, healthcare resource utilization, and decision-making. Models that can guide personalized interventions, improve prognostic accuracy, and enhance patient care are more likely to be adopted in clinical practice.

By conducting a thorough comparative analysis based on these key considerations, researchers and healthcare practitioners can gain a deeper understanding of the strengths and limitations of different predictive models and parameters for hepatic steatosis progression. This analysis can inform the selection of the most appropriate predictive tools for specific clinical contexts, ultimately leading to more effective and personalized management of hepatic steatosis and improved patient outcomes.

## 5. Clinical Implications and Future Directions

### 5.1. The Clinical Implications of Using Evolutive Models, Algorithms, and Predictive Parameters in the Management of Hepatic Steatosis

The clinical implications of utilizing evolutive models, algorithms, and predictive parameters in the management of hepatic steatosis are significant and encompass various aspects of patient care, risk stratification, and treatment decision-making. These implications extend to disease monitoring, prognostication, and personalized intervention strategies, thereby contributing to improved clinical outcomes and patient well-being.

#### 5.1.1. Personalized Risk Stratification

The integration of evolutive models, algorithms, and predictive parameters enables the personalized risk stratification of individuals with hepatic steatosis. By considering a comprehensive set of biomarkers, imaging findings, and clinical data, these approaches can aid in identifying patients at higher risk of disease progression, complications, and adverse outcomes. As a result, clinicians can tailor their management strategies and interventions to address the specific needs and risks of each patient.

#### 5.1.2. Early Detection and Intervention

Evolutive models and predictive parameters offer the potential to facilitate the early detection of hepatic steatosis progression. By identifying subtle changes in biomarkers, imaging features, or predictive scores, clinicians can intervene at an earlier stage, potentially preventing the development of advanced liver disease and associated complications. This early intervention may involve lifestyle modifications, pharmacological interventions, or the closer monitoring of disease progression.

#### 5.1.3. Treatment Decision Support

The use of evolutive models and predictive parameters can support treatment decision-making in individuals with hepatic steatosis. By providing insights into the likelihood of disease progression, response to specific interventions, or the efficacy of lifestyle modifications, these tools can assist healthcare providers in selecting appropriate treatment modalities and optimizing therapeutic approaches for individual patients.

#### 5.1.4. Monitoring Disease Progression

Evolutive models and predictive parameters facilitate the continuous monitoring of disease progression in individuals with hepatic steatosis. By incorporating longitudinal data and dynamic changes in predictive scores or biomarker levels, clinicians can track the evolution of the disease, assess treatment response, and adjust management strategies accordingly. This proactive monitoring approach may enhance disease control and reduce the risk of adverse outcomes.

#### 5.1.5. Clinical Trial Design and Drug Development

The integration of evolutive models, algorithms, and predictive parameters can impact the design of clinical trials and the development of targeted therapeutics for hepatic steatosis. By identifying patient subgroups with distinct disease trajectories or treatment responses, these tools can support the stratification of participants in clinical trials, the selection of appropriate endpoints, and the evaluation of treatment efficacy. Furthermore, predictive parameters may aid in identifying potential therapeutic targets and biomarkers for drug development.

### 5.2. Future Directions

#### 5.2.1. Integration of Omics Data

Future research may focus on integrating omics data, including genomics, transcriptomics, proteomics, and metabolomics, into evolutive models and predictive algorithms for hepatic steatosis. This multidimensional approach could yield novel biomarkers, mechanistic insights, and personalized risk predictions, enhancing the precision and clinical utility of predictive parameters.

#### 5.2.2. Patient-Centered Outcomes Research

Future studies may emphasize patient-centered outcomes research to align evolutive models and predictive parameters with patient preferences, quality of life measures, and individualized treatment goals. By incorporating patient-reported outcomes and preferences into predictive algorithms, clinicians can deliver more patient-centric care and personalized interventions tailored to the needs and values of each individual.

#### 5.2.3. Real-Time Decision Support Systems

The development of real-time decision support systems that integrate evolutive models and predictive parameters into clinical practice could enhance the proactive management of hepatic steatosis. Such systems may provide clinicians with dynamic risk assessments, treatment recommendations, and prognostic insights, facilitating timely interventions and personalized care delivery.

In conclusion, the integration of evolutive models, algorithms, and predictive parameters holds substantial clinical implications for the management of hepatic steatosis. These approaches offer opportunities for personalized risk stratification, early detection, treatment decision support, disease monitoring and advancements in clinical trial design. Furthermore, future directions in hepatic steatosis research may involve the integration of omics data, patient-centered outcomes research, and real-time decision support systems to further enhance the precision and clinical utility of predictive parameters in this evolving field.

### 5.3. The Potential Impact on Patient Outcomes, Risk Stratification, and the Development of Personalized Treatment Strategies

The potential impact of using evolutive models, algorithms, and predictive parameters in the management of hepatic steatosis on patient outcomes, risk stratification, and the development of personalized treatment strategies is significant and can lead to tangible improvements in clinical care and patient well-being.

#### 5.3.1. Patient Outcomes

By incorporating evolutive models and predictive parameters into clinical practice, several potential impacts on patient outcomes can be anticipated.

##### Improved Disease Management

The use of predictive algorithms and models can lead to more proactive and personalized disease management, potentially resulting in the improved control of hepatic steatosis and related complications. The early detection of disease progression, coupled with personalized interventions, has the potential to mitigate the development of advanced liver disease and improve overall patient outcomes.

##### Reduced Disease Burden

Personalized risk stratification based on evolutive models can identify individuals at higher risk of disease progression, allowing for targeted interventions. This approach has the potential to reduce the overall burden of hepatic steatosis-related complications, including advanced liver disease, cirrhosis, and hepatocellular carcinoma, leading to better long-term outcomes for patients.

##### Enhanced Quality of Life

Tailoring treatment strategies based on predictive parameters can contribute to the improvement of patients’ quality of life by minimizing the impact of hepatic steatosis on daily functioning, reducing symptom burden, and potentially avoiding the need for more invasive interventions and hospitalizations.

#### 5.3.2. Risk Stratification

Evolutive models and predictive parameters can significantly impact risk stratification in the following ways.

##### Precision Medicine Approaches

The use of predictive algorithms can enable the identification of patient subgroups with distinct disease trajectories and treatment responses, allowing for more precise risk stratification. This approach may facilitate the delivery of personalized interventions tailored to the specific needs and risks of each patient, improving overall risk stratification strategies.

##### Early Identification of High-Risk Patients

Evolutive models provide the potential to identify patients at higher risk of disease progression at an earlier stage, enabling timely risk stratification and intervention. This early identification can lead to more effective risk mitigation strategies and prevent the escalation of hepatic steatosis-related complications.

##### Tailored Monitoring and Surveillance

Personalized risk stratification based on evolutive models can guide the intensity and frequency of monitoring and surveillance for individuals with hepatic steatosis. This tailored approach ensures that patients at higher risk receive closer monitoring, while low-risk individuals may undergo less frequent surveillance, optimizing resource allocation and patient care.

#### 5.3.3. Development of Personalized Treatment Strategies

The integration of evolutive models and predictive parameters can revolutionize the development of personalized treatment strategies in the following ways.

##### Targeted Interventions

Predictive algorithms can guide the selection of targeted interventions based on an individual’s predicted disease progression and treatment response. This personalized approach may include lifestyle modifications, pharmacological interventions, and behavioral interventions tailored to each patient’s specific risk profile and disease trajectory.

##### Optimization of Therapeutic Outcomes

Tailoring treatment strategies based on predictive parameters can optimize therapeutic outcomes by aligning interventions with the predicted course of hepatic steatosis in individual patients. This approach may enhance the efficacy of treatment, potentially leading to better disease control and improved patient outcomes.

##### Individualized Risk-Benefit Assessment

Evolutive models can support an individualized risk–benefit assessment of treatment options, considering the predicted disease progression, potential treatment responses, and the likelihood of adverse effects. This personalized approach ensures that treatment strategies are aligned with each patient’s unique risk profile and treatment goals.

By leveraging evolutive models and predictive parameters, the development of personalized treatment strategies for hepatic steatosis has the potential to significantly enhance patient outcomes, improve risk stratification, and optimize therapeutic approaches. These advancements in personalized medicine can lead to more effective disease management, a reduced disease burden, and a better quality of life for individuals affected by hepatic steatosis. Furthermore, by tailoring interventions based on an individual’s predicted disease progression and treatment response, personalized treatment strategies can optimize therapeutic outcomes and minimize the impact of hepatic steatosis on patients’ lives.

### 5.4. Considering Future Directions for Research in This Area, including the Need for Prospective Studies, Validation of Predictive Models, and Integration of Novel Technologies

Future research in the management of hepatic steatosis will benefit from the comprehensive exploration and implementation of prospective studies, the validation of predictive models, and the integration of novel technologies. These endeavors will be crucial for advancing the field, refining predictive capabilities, and enhancing the personalized management of hepatic steatosis. Key future directions for research in this area include the following.

#### 5.4.1. Prospective Studies

Prospective studies are essential for elucidating the natural history of hepatic steatosis, identifying robust predictive parameters, and validating the accuracy of evolutive models and algorithms. By following cohorts of individuals over time, prospective studies can provide valuable insights into disease progression, risk factors, and the trajectory of hepatic steatosis. These studies can also establish the long-term prognostic significance of predictive parameters and models, facilitating the development of evidence-based predictive tools for clinical practice.

#### 5.4.2. Validation of Predictive Models

The validation of predictive models for hepatic steatosis is critical for ensuring their accuracy, reliability, and clinical utility. Future research efforts should focus on comprehensive validation studies that assess the performance of evolutive models and algorithms in diverse patient populations, accounting for variations in disease etiology, comorbidities, and demographic factors. Rigorous validation efforts will enable the identification of robust predictive models that can accurately stratify patients based on their risk of disease progression, inform personalized treatment strategies, and guide clinical decision-making.

#### 5.4.3. Integration of Novel Technologies

The integration of novel technologies holds promise for advancing the diagnosis, monitoring, and prediction of hepatic steatosis. Future research should focus on leveraging advanced imaging modalities, such as quantitative MRI techniques, magnetic resonance elastography, and spectroscopy, to refine the assessment of hepatic steatosis and its progression. Additionally, the exploration of novel non-invasive biomarkers, omics-based approaches, and digital health technologies can expand the repertoire of predictive parameters and enhance the precision of evolutive models. Integrating these innovative technologies into research protocols and clinical practice will enable the development of comprehensive, multi-modal predictive tools for hepatic steatosis.

#### 5.4.4. Personalized Predictive Algorithms

The development of personalized predictive algorithms that consider individual patient characteristics, genetic factors, and metabolic parameters represents a promising avenue for future research. By incorporating a wide range of patient-specific data, including genetic polymorphisms, metabolic profiles, lifestyle factors, and demographic variables, personalized predictive algorithms can provide tailored risk assessments and treatment recommendations. Advancing research efforts in this direction will necessitate interdisciplinary collaborations, integrating expertise from genomics, metabolomics, bioinformatics, and digital health to develop comprehensive, individualized predictive algorithms for hepatic steatosis.

#### 5.4.5. Longitudinal Data Analysis

Longitudinal data analysis approaches, including the utilization of machine learning and deep learning techniques, can offer valuable insights into the dynamic progression of hepatic steatosis. By leveraging longitudinal data from diverse cohorts, these advanced analytical methods can reveal complex patterns of disease evolution, predict individualized disease trajectories, and identify novel predictive features that may inform personalized management strategies. Future research should prioritize the application of longitudinal data analysis to unravel the intricate dynamics of hepatic steatosis progression and refine predictive models for clinical use.

#### 5.4.6. Collaborative Consortia and Data Sharing

Establishing collaborative consortia and promoting data sharing initiatives within the research community will be instrumental in advancing the field of hepatic steatosis prediction and management. By fostering multi-center collaborations, pooling diverse datasets, and sharing comprehensive repositories of clinical and research data, the scientific community can accelerate the development and validation of predictive models, enabling robust, generalizable, and clinically relevant tools for hepatic steatosis prediction. These collaborative efforts can also facilitate the standardization of predictive parameters and risk stratification methodologies, promoting consistency and comparability across studies.

#### 5.4.7. Ethical and Regulatory Considerations

As research in hepatic steatosis prediction and personalized management advances, it is essential to address ethical and regulatory considerations related to the use of predictive algorithms in clinical practice. Future research should focus on evaluating the ethical implications of predictive models, ensuring patient privacy, transparency, and informed consent in the implementation of predictive tools. Additionally, regulatory frameworks should be designed to ensure the safety, accuracy, and ethical use of predictive algorithms in healthcare settings, promoting the responsible and equitable application of personalized predictive models for hepatic steatosis.

In conclusion, future research in the field of hepatic steatosis prediction and personalized management should prioritize prospective studies, the validation of predictive models, the integration of novel technologies, the development of personalized predictive algorithms, longitudinal data analysis, collaborative consortia, data sharing initiatives, and ethical and regulatory considerations. By advancing research efforts in these directions, the scientific community can drive the development of robust, accurate, and clinically relevant predictive tools for hepatic steatosis, ultimately enhancing personalized management strategies and improving patient outcomes.

### 5.5. Comparative Analysis

#### 5.5.1. Comparing Different Parameters That Have Been Proposed in the Literature for Assessing Hepatic Steatosis Progression

Various parameters have been proposed in the literature for assessing the progression of hepatic steatosis. These approaches aim to provide accurate and timely predictions of disease progression, enabling personalized management strategies. Here is a comparative analysis of some of the key predictive models, algorithms, and parameters proposed in the literature.

##### Imaging-Based Models

Magnetic Resonance Imaging (MRI)-Based Models: MRI-based techniques, such as proton density fat fraction (PDFF) and magnetic resonance elastography (MRE), have been used to assess hepatic steatosis progression. These non-invasive imaging modalities enable the quantification of hepatic fat content and stiffness, allowing for the longitudinal monitoring of disease progression [47].

Computed Tomography (CT) Imaging-Based Models: CT imaging has also been utilized to assess hepatic steatosis progression [48]. CT attenuation measurements and texture analysis have been employed to predict the severity and progression of hepatic steatosis.

##### Non-Invasive Biomarkers

Serum Biomarkers: Several serum biomarkers, including markers of lipid metabolism (e.g., adiponectin, leptin), liver injury (e.g., AST to ALT ratio, cytokeratin-18 fragments), and inflammation (e.g., C-reactive protein), have been investigated as potential predictors of hepatic steatosis progression [49]. These biomarkers provide insights into the metabolic and inflammatory processes associated with disease progression.

Genetic Biomarkers: Genetic variants associated with hepatic steatosis, such as PNPLA3 and TM6SF2 polymorphisms, have been studied as predictors of disease progression [50]. Genetic biomarkers offer valuable information about the underlying genetic predisposition to hepatic steatosis progression.

##### Computational Models and Algorithms

Machine Learning-Based Models: Machine learning algorithms, including support vector machines (SVM), random forests, and neural networks, have been applied to predict hepatic steatosis progression [51]. These models utilize a combination of clinical, imaging, and biomarker data to generate personalized predictions of disease progression.

Risk Stratification Models: Risk scores and stratification models, such as the NAFLD fibrosis score and FIB-4 index, have been developed to assess the risk of progression to advanced fibrosis in patients with hepatic steatosis [52]. These models integrate clinical and laboratory data to identify individuals at higher risk of disease progression.

##### Histological Parameters

Histological Scoring Systems: Histological scoring systems, such as the NAFLD Activity Score (NAS) and the Fibrosis-4 (F4) score, have been used to assess the severity of hepatic steatosis and the risk of progression to advanced fibrosis [53]. These scoring systems provide valuable insights into histological changes associated with disease progression.

In comparing these approaches, it is important to consider their strengths and limitations. Imaging-based models, such as MRI and CT, offer non-invasive and quantitative assessments of hepatic steatosis progression, but they may be limited by accessibility and cost. Non-invasive biomarkers provide valuable insights into the molecular and genetic factors associated with disease progression, but their predictive accuracy may vary across different patient populations. Computational models and algorithms, including machine learning-based approaches, have the potential to integrate diverse data sources and generate personalized predictions, but they may require robust validation in clinical practice. Histological parameters, while providing direct insights into disease pathology, are limited by the invasiveness of liver biopsy and the potential for sampling variability.

Overall, the selection of predictive models, algorithms, and parameters for assessing hepatic steatosis progression should take into account the specific clinical context, the available resources, and the need for personalized and reliable predictions. Integrating multiple modalities, including imaging, biomarkers, computational models, and histological parameters, may offer a comprehensive approach to predicting hepatic steatosis progression, enabling tailored management strategies and improved patient outcomes.

### 5.6. Evaluating the Strengths and Weaknesses of Each Approach, Highlighting the Potential for Integration or Combination of Multiple Models to Improve Predictive Accuracy

Evaluating the strengths and weaknesses of each approach for assessing hepatic steatosis progression is crucial in understanding their potential and limitations. Additionally, considering the potential for integrating or combining multiple models to improve predictive accuracy is essential for developing comprehensive and reliable predictive strategies. We have analyzed the strengths and weaknesses of each approach and explored the potential for integration or combination of multiple models to enhance predictive accuracy.

#### 5.6.1. Imaging-Based Models

Strengths:

– Provide non-invasive and quantitative assessment of hepatic steatosis.
– Enable longitudinal monitoring of disease progression.
– Offer insights into hepatic fat content and tissue stiffness.

Weaknesses:

– Limited accessibility and cost of advanced imaging modalities.
– May not capture molecular or genetic factors associated with disease progression.

Integration Potential:

– Combining MRI and CT imaging data with molecular biomarkers could offer a more comprehensive assessment, capturing both structural changes and underlying molecular mechanisms.

#### 5.6.2. Non-Invasive Biomarkers

Strengths:

– Reflect metabolic, inflammatory, and genetic factors associated with hepatic steatosis progression.
– Easily accessible and can be measured through routine blood tests.

Weaknesses:

– Variable predictive accuracy across different patient populations.
– Limited ability to capture structural changes in the liver.

Integration Potential:

– Integration of serum biomarkers with imaging data could offer a multi-dimensional view of disease progression, capturing both molecular and structural changes in the liver.

#### 5.6.3. Computational Models and Algorithms

Strengths:

– Ability to integrate diverse data sources, including imaging, biomarkers, and clinical variables.
– Potential for generating personalized predictions based on individual patient data.

Weaknesses:

– Require robust validation in clinical practice.
– Interpretability and transparency of complex machine learning models may be limited.

Integration Potential:

– Integration of machine learning-based predictions with histological parameters could provide a comprehensive understanding of disease progression, combining non-invasive assessments with direct histological insights.

#### 5.6.4. Histological Parameters:

Strengths:

– Provide direct insights into disease pathology and severity.
– Can capture histological changes associated with disease progression.

Weaknesses:

– Invasive nature of liver biopsy and associated sampling variability.
– Limited ability to perform longitudinal monitoring due to invasiveness.

Integration Potential:

– Combining histological scoring systems with non-invasive imaging and biomarker data could provide a more holistic view of hepatic steatosis progression, incorporating both structural and molecular assessments.

Integration Potential:

– Integrating data from multiple models, including imaging, biomarkers, computational predictions, and histological parameters, holds significant promise in improving predictive accuracy for hepatic steatosis progression.
– An integrated approach could leverage the strengths of each model while compensating for individual weaknesses, offering a more comprehensive and multi-dimensional assessment of disease progression.

For example, combining non-invasive imaging data with molecular biomarkers and computational predictions could provide a more thorough understanding of disease progression, enabling personalized risk stratification and treatment planning. Furthermore, longitudinal monitoring using imaging-based models could be complemented by periodic assessments of biomarkers and computational predictions to track changes in disease severity and response to interventions over time.

In conclusion, the integration or combination of multiple predictive models, algorithms and parameters has the potential to enhance the accuracy and reliability of predicting hepatic steatosis progression. By leveraging the strengths of each approach and addressing their respective weaknesses, an integrated approach could provide a more comprehensive assessment, leading to improved risk stratification, the early detection of disease progression, and personalized management strategies for individuals with hepatic steatosis.

### 5.7. Ethical and Social Implications

#### 5.7.1. Considering the Ethical Considerations and Social Implications Associated with the Implementation of Evolutive Models and Predictive Parameters for Hepatic Steatosis

The implementation of evolutive models and predictive parameters for hepatic steatosis raises several ethical considerations and social implications that require thoughtful assessment and mitigation strategies. These considerations are vital to ensure the responsible and equitable application of predictive tools in clinical practice. We have delved into some of the ethical and social implications associated with the implementation of evolutive models and predictive parameters for hepatic steatosis.

##### Privacy and Data Protection

– Ethical Considerations: The use of patient data, including medical records, genetic information, and imaging data, to develop and validate predictive models raises concerns regarding patient privacy and data protection. Ensuring informed consent, data anonymization, and stringent security measures to safeguard sensitive patient information is essential to maintain patient trust and uphold ethical standards.
– Social Implications: Patients and the wider community may express apprehension about the use of their health data for predictive modeling. Transparency about data usage, protection measures, and explicit consent mechanisms is crucial to allay concerns and foster trust in the healthcare system.

##### Equity and Access

– Ethical Considerations: The equitable access to predictive models and personalized risk assessments is critical. Issues of healthcare disparities, particularly regarding access to advanced imaging modalities and biomarker testing, must be addressed to ensure that predictive tools do not exacerbate existing healthcare inequities.
– Social Implications: Disparities in access to predictive technologies could perpetuate healthcare inequalities, leading to differential outcomes for individuals with hepatic steatosis. Efforts to promote equitable access and address disparities in healthcare resources are essential to mitigate these social implications.

##### Informed Decision-Making

– Ethical Considerations: Healthcare providers must uphold the principles of informed consent and shared decision-making when integrating predictive parameters into clinical practice. Patients should be educated about the implications, limitations, and potential benefits of predictive modeling to make informed decisions about their care.
– Social Implications: Empowering patients to understand and engage with predictive models can enhance patient autonomy and lead to more collaborative and personalized healthcare interactions. However, ensuring that patients are not unduly influenced or overwhelmed by predictive information is crucial to mitigate potential harms related to anxiety and unnecessary medical interventions.

##### Algorithmic Bias and Transparency

– Ethical Considerations: The development and implementation of evolutive models and algorithms should address potential biases related to race, gender, socioeconomic status, and other demographic factors. Transparency about model development, validation processes, and potential limitations is essential to ensure ethical and fair application.
– Social Implications: Unmitigated algorithmic bias and lack of transparency in predictive modeling can perpetuate healthcare disparities and undermine trust in healthcare systems. Efforts to promote fairness, accountability, and transparency in the development and deployment of predictive parameters are essential to mitigate these social implications.

##### Impact on Clinical Practice

– Ethical Considerations: The integration of predictive parameters into clinical decision-making raises challenges related to the appropriate interpretation and use of predictive information. Healthcare providers’ ethical responsibilities include ensuring that predictive models supplement, rather than replace, clinical judgment and holistic patient care.
– Social Implications: The appropriate integration of predictive parameters can enhance the precision and personalization of healthcare interventions. However, concerns about overreliance on predictive tools, potential diagnostic labeling, and impacts on the patient–provider relationship merit attention to prevent unintended negative social consequences.

Addressing these ethical considerations and the social implications associated with the implementation of evolutive models and predictive parameters for hepatic steatosis is essential to foster the responsible and equitable use of predictive technologies in clinical practice. Ethical guidelines, patient engagement strategies, regulatory oversight, and the ongoing assessment of social impacts are crucial components of ensuring that the implementation of predictive parameters prioritizes patient well-being, fairness, and trust within healthcare systems.

### 5.8. Discussing Issues Related to Data Privacy, Equity in Access to Advanced Diagnostic Technologies, and the Potential Impact on Healthcare Disparities

#### 5.8.1. Data Privacy

The implementation of evolutive models and predictive parameters for hepatic steatosis involves the utilization of diverse patient data, including genetic information, medical records, and imaging data. Ensuring data privacy and protection is crucial to maintain patient trust and uphold ethical standards. The ethical considerations related to data privacy encompass the following.

##### Informed Consent

Healthcare providers must prioritize obtaining informed consent from patients regarding the use of their data for the development and validation of predictive models. Patients should be educated about how their data will be used, the potential benefits and limitations of predictive modeling, and measures taken to protect their privacy.

##### Data Anonymization

To mitigate privacy risks, strict protocols for data anonymization should be implemented to remove personally identifiable information from datasets used for model development and validation. This approach can help prevent the identification of individual patients through their data contributions to the predictive models.

##### Security Measures

Robust security measures, including encryption, access controls, and secure data storage, must be in place to safeguard sensitive patient information. By implementing strong cybersecurity practices, healthcare organizations can mitigate the risk of data breaches and unauthorized access to patient data.

#### 5.8.2. Equity in Access to Advanced Diagnostic Technologies

The equitable access to advanced diagnostic technologies utilized in the development and implementation of predictive parameters for hepatic steatosis is a critical concern. Issues related to healthcare disparities, including access to imaging modalities and biomarker testing, must be addressed to ensure that predictive tools do not exacerbate existing healthcare inequities. The ethical and social considerations related to equity in access encompass the following.

##### Healthcare Disparities

Disparities in access to advanced diagnostic technologies can lead to differential outcomes for individuals with hepatic steatosis, perpetuating healthcare inequalities. Certain populations, such as those from lower socioeconomic backgrounds or underserved communities, may face challenges in accessing advanced diagnostic technologies, potentially limiting their access to personalized risk assessments and interventions.

##### Resource Allocation

Efforts to promote equitable access and address disparities in healthcare resources are essential. Healthcare organizations and policymakers should prioritize resource allocation to ensure that individuals from all demographic groups have access to advanced diagnostic technologies necessary for the development and implementation of predictive parameters for hepatic steatosis.

#### 5.8.3. Healthcare Disparities and Predictive Modeling

The potential impact of predictive modeling on healthcare disparities is a significant consideration. Unaddressed disparities in access to advanced diagnostic technologies and predictive modeling tools can lead to differential outcomes based on socioeconomic status, geographic location, or other demographic factors. The ethical and social implications related to the potential impact on healthcare disparities encompass the following.

##### Mitigating Bias

The development and implementation of predictive models should address potential biases related to race, gender, socioeconomic status, and other demographic factors. Equitable representation and the consideration of diverse patient populations in the development and validation of predictive parameters can help mitigate biases and ensure that the models are applicable to a wide range of individuals.

##### Transparency and Accountability

Transparency about the development, validation processes, and potential limitations of predictive models is crucial to mitigate potential impacts on healthcare disparities. Healthcare organizations and researchers should strive for accountability and transparency in the development and deployment of predictive parameters, thereby promoting a fair and unbiased application of these tools across diverse patient populations.

Addressing these issues related to data privacy, equity in access to advanced diagnostic technologies, and the potential impact on healthcare disparities is essential to fostering the responsible and equitable use of predictive parameters for hepatic steatosis. By prioritizing patient privacy, addressing healthcare disparities, and promoting fairness and accountability in the development and implementation of predictive models, healthcare organizations can strive to ensure that the benefits of predictive technologies are accessible to all individuals irrespective of their background or socioeconomic status.

## 6. Conclusions

In conclusion, our article highlights the importance of advancing predictive models and algorithms for the progression of hepatic steatosis, emphasizing key findings and implications. The utilization of evolutive models, algorithms, and predictive parameters has the potential to revolutionize the management of hepatic steatosis by improving risk stratification, personalizing treatment strategies, and enhancing patient outcomes. The manuscript has elucidated the following key findings and implications:

### 6.1. Key Findings

#### 6.1.1. Diverse Approaches

Various approaches, including imaging-based quantification, non-invasive biomarkers, computational models, and histological parameters, offer unique insights into the progression of hepatic steatosis.

#### 6.1.2. Predictive Potential

These approaches demonstrate strong potential for predicting disease progression, enabling personalized risk assessments, and informing tailored interventions.

#### 6.1.3. Integration Opportunities

The integration of multiple models and data sources holds promise for improving predictive accuracy and offering a comprehensive understanding of hepatic steatosis progression.

### 6.2. Implications

#### 6.2.1. Personalized Risk Assessment

Advancing predictive models facilitates the development of personalized risk assessments for individuals with hepatic steatosis, enabling early detection and targeted interventions.

#### 6.2.2. Enhanced Treatment Strategies

The utilization of predictive algorithms supports the development of tailored treatment strategies, ensuring that interventions align with individual patient needs and disease trajectories.

#### 6.2.3. Healthcare Disparities

Addressing issues of data privacy and equity in access to advanced diagnostic technologies is paramount to ensure that predictive models do not exacerbate healthcare disparities and promote fair and equitable use across diverse patient populations.

The implications of advancing predictive models and algorithms for the progression of hepatic steatosis are far-reaching and encompass improved patient outcomes, optimized resource allocation, and the potential to mitigate healthcare disparities. By leveraging predictive tools, healthcare providers can offer personalized risk assessments, target interventions, and improve the overall management of hepatic steatosis. Furthermore, the integration of diverse models and data sources has the potential to enhance predictive accuracy and support the development of personalized treatment strategies.

Ultimately, the advancement of predictive models and algorithms for the progression of hepatic steatosis represents a critical frontier in personalized medicine and precision healthcare. Through continued research, validation, and ethical considerations related to data privacy and healthcare disparities, these predictive tools have the potential to transform the management of hepatic steatosis and improve patient outcomes, paving the way for a more personalized and effective approach to hepatic steatosis management.

## Data Availability

The data presented in this study are available in article.
